# The Effect of Metformin and GANT61 Combinations on the Radiosensitivity of Prostate Cancer Cells

**DOI:** 10.3390/ijms18020399

**Published:** 2017-02-13

**Authors:** Annelies Gonnissen, Sofie Isebaert, Chad M. McKee, Ruth J. Muschel, Karin Haustermans

**Affiliations:** 1 Laboratory of Experimental Radiotherapy, Department of Oncology, KU Leuven—University of Leuven, 3000 Leuven, Belgium; annelies.gonnissen@kuleuven.be (A.G.); karin.haustermans@uzleuven.be (K.H.); 2 Department of Radiation Oncology, University Hospitals Leuven, 3000 Leuven, Belgium; 3 Department of Oncology, CRUK/MRC Oxford Institute for Radiation Oncology, University of Oxford, Oxford OX3 7DQ, UK; chad.mckee@oncology.ox.ac.uk (C.M.M.); ruth.muschel@oncology.ox.ac.uk (R.J.M.)

**Keywords:** prostate cancer, metformin, hedgehog pathway, radiosensitization, xenograft mouse model

## Abstract

The anti-diabetes drug metformin has been shown to have anti-neoplastic effects in several tumor models through its effects on energy metabolism and protein synthesis. Recent studies show that metformin also targets Hedgehog (Hh) signaling, a developmental pathway re-activated in several tumor types, including prostate cancer (PCa). Furthermore, we and others have shown that Hh signaling is an important target for radiosensitization. Here, we evaluated the combination of metformin and the Hh inhibitor GANT61 (GLI-ANTagonist 61) with or without ionizing radiation in three PCa cell lines (PC3, DU145, 22Rv1). The effect on proliferation, radiosensitivity, apoptosis, cell cycle distribution, reactive oxygen species production, DNA repair, gene and protein expression was investigated. Furthermore, this treatment combination was also assessed in vivo. Metformin was shown to interact with Hh signaling by inhibiting the effector protein glioma-associated oncogene homolog 1 (GLI1) in PCa cells both in vitro and in vivo. The combination of metformin and GANT61 significantly inhibited PCa cell growth in vitro and enhanced the radiation response of 22Rv1 cells compared to either single agent. Nevertheless, neither the growth inhibitory effect nor the radiosensitization effect of the combination treatment observed in vitro was seen in vivo. Although the interaction between metformin and Hh signaling seems to be promising from a therapeutic point of view in vitro, more research is needed when implementing this combination strategy in vivo.

## 1. Introduction

The biguanide metformin, commonly used for the treatment of patients with type 2 diabetes, has been associated with a decreased incidence and mortality in several tumor types, including prostate cancer (PCa) [1,2,3]. Metformin has also been shown to have anti-neoplastic effects [4], potentially due to its effects on energy metabolism and protein synthesis by activation of adenosine monophosphate (AMP)-activated protein kinase (AMPK) signaling and inhibition of the mammalian target of rapamycin (mTOR) [5,6,7]. Additionally, several preclinical studies have shown that metformin can increase the effect of radiotherapy [8,9,10,11,12]. The mechanism of these radiosensitizing effects are not completely understood but include reoxygenation of hypoxic regions [9], impairment of DNA damage response [11] and induction of reactive oxygen species (ROS) [12]. The radiosensitizing effect of metformin has been shown to be highly context-dependent and more research is needed to identify the appropriate treatment conditions and cell populations to induce radiosensitization by metformin.

Some recent studies have identified the Hedgehog (Hh) signaling pathway as a novel target of metformin [13,14]. This pathway is a potential candidate for anti-cancer treatment, since several tumor types are known to have deregulated Hh signaling, including PCa [15,16,17,18]. Of note, metformin has been shown to inhibit Hh signaling in breast [13] and pancreatic [14] cancer cells.

We have previously shown that inhibition of Hh signaling at the level of the transcription factors using the small molecule inhibitor GANT61 (GLI-ANTagonist 61) is highly effective in PCa cells and increased radiosensitivity both in vitro and in vivo [19].

In this study, the link between AMPK and Hh signaling was explored in several PCa cell lines. Additionally, we investigated whether simultaneous targeting of both pathways with metformin and GANT61, respectively, could enhance PCa cytotoxicity and/or radiosensitivity both in vitro and in vivo.

## 2. Results

### 2.1. Metformin Decreases Cell Growth and Survival of Prostate Cancer (PCa) Cells

**Figure 1:**
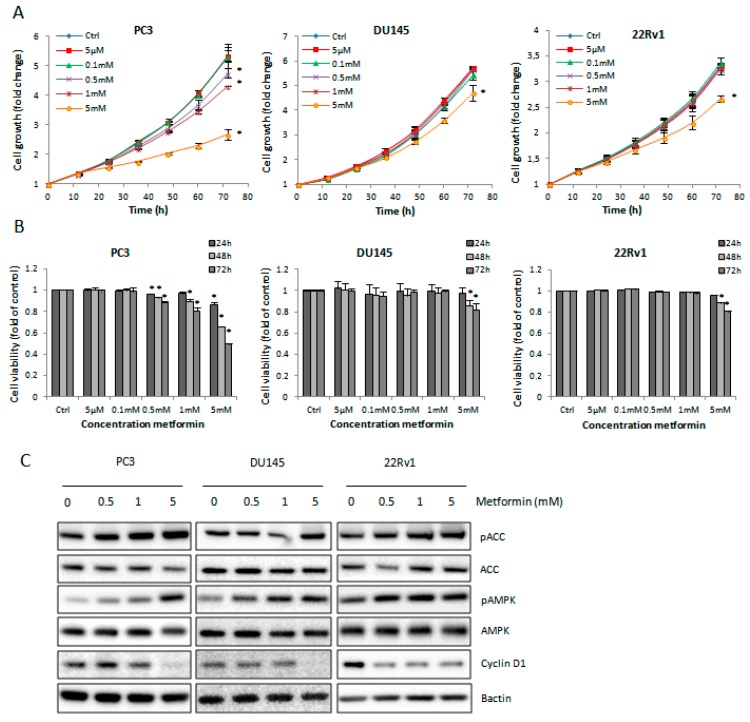
Effect of metformin on prostate cancer (PCa) cells. (**A**) Cell growth and (**B**) cell viability after metformin treatment. Means ± standard error of means (SEM) of three independent experiments. * *p* < 0.05 vs. control; (**C**) Protein expression of the downstream signaling molecules after 72-h metformin treatment. ACC, Acetyl-CoA carboxylase; AMPK, adenosine monophosphate (AMP)-activated protein kinase; pACC, phospho-Acetyl-CoA carboxylase; pAMPK, phospho-Adenosine monophosphate (AMP)-activated protein kinase.

Metformin significantly decreased PCa cell growth in a time- and dose-dependent manner (Figure 1A). A high dose of metformin (5 mM) was able to induce a significant decrease in cell survival in all cell lines (Figure 1B). The PC3 cells were the most sensitive with a half maximal inhibitory concentration (IC_50_) value around 5 mM, whereas the IC_50_ in 22Rv1 and DU145 was between 10–20 mM metformin (Figure S1).

In line with this, cyclin D1 protein expression was drastically decreased after treatment with 5 mM metformin, especially in the rapidly proliferating PC3 and DU145 cell lines (Figure 1C). Additionally, metformin activated its downstream signaling components AMPK and Acetyl-CoA carboxylase (ACC) in a dose-dependent manner in all PCa cell lines (Figure 1C).

### 2.2. Metformin Increases Radiosensitivity of PCa Cells Independent of Adenosine Monophosphate (AMP)-Activated Protein Kinase (AMPK) Activation

**Figure 2:**
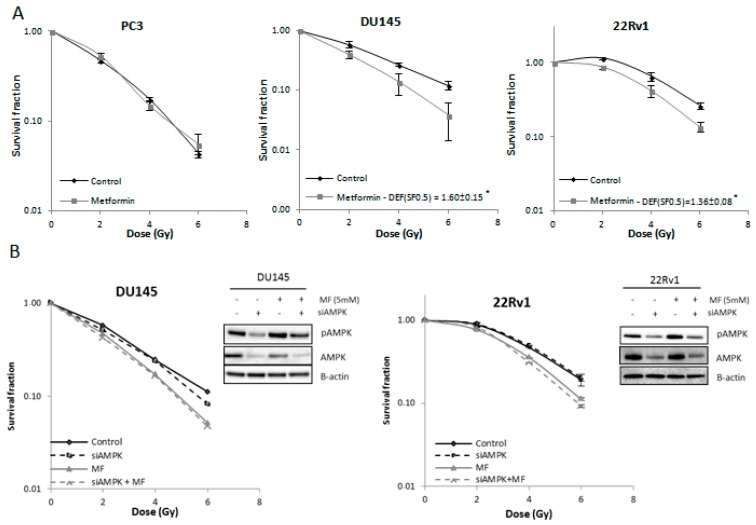
Effect of metformin (MF) on radiosensitivity of PCa cells. (**A**) Clonogenic survival after 72-h treatment with metformin (5 mM) prior to/during ionizing radiation (IR); (**B**) Clonogenic survival of DU145 and 22Rv1 cells transfected with AMPK silencing RNA (siRNA) and 72-h treatment with metformin (5 mM) prior to/during IR. Knockdown was verified with western blotting. Means ± SEM of three independent experiments. * *p* < 0.05 vs. control. DEF: dose-enhancement factor.

Metformin (5 mM) increased radiosensitivity of DU145 and 22Rv1 cells with a dose-enhancement factor (DEF) of 1.6 ± 0.15 (*p* < 0.05) and 1.36 ± 0.08 (*p* < 0.05) respectively. In contrast, the radiosensitivity of PC3 cells was not affected by metformin (Figure 2A). To evaluate the role of AMPK in the metformin-induced radiosensitization effect in the DU145 and 22Rv1 cells, AMPK was silenced by means of silencing RNA (siRNA). Downregulation of (phospho)AMPK did not affect the intrinsic radiosensitivity of either cell line nor did it change the metformin-induced radiosensitization (Figure 2B).

### 2.3. Metformin Regulates Hedgehog Signaling in an AMPK-Dependent Manner

**Figure 3:**
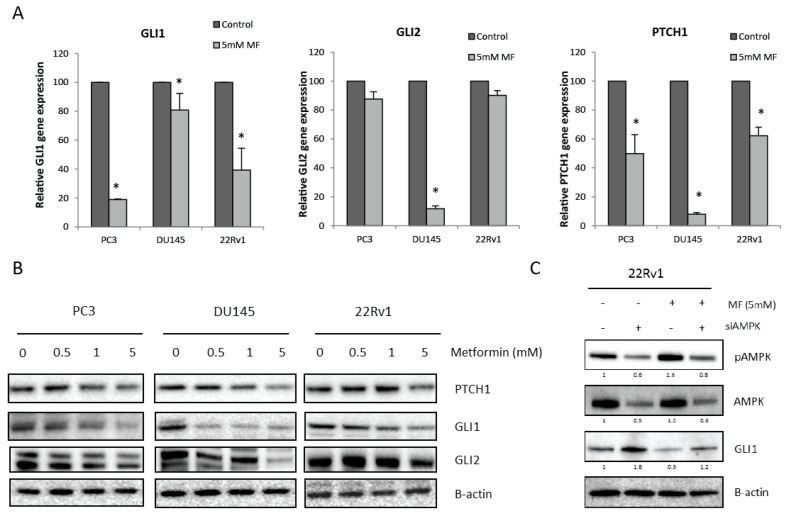
Link between metformin and Hedgehog signaling. (**A**) *GLI1*, *GLI2* and *PTCH1* gene expression after 72-h metformin treatment. Means ± SEM of two independent experiments. * *p* < 0.05 vs. control; (**B**) PTCH1, GLI1 and GLI2 protein expression after 72-h metformin treatment; (**C**) (p)AMPK protein and GLI1 expression in 22Rv1 cells transfected with AMPK siRNA and treated with metformin (5 mM) 72-h prior to protein lysis. GLI1, glioma-associated oncogene homolog 1; GLI2, glioma-associated oncogene homolog 2; PTCH1, patched 1.

Next, we investigated if there was a link between metformin and Hh signaling in PCa cells. Indeed, metformin (5 mM) significantly decreased glioma-associated oncogene homolog 1 (*GLI1*) and patched 1 (*PTCH1*) gene and protein expression in all cell lines (Figure 3A,B). Although metformin only significantly decreased glioma-associated oncogene homolog 2 (*GLI2*) gene expression in the DU145 cells, we did observe decreased GLI2 protein expression after metformin treatment in all PCa cell lines (Figure 3A,B). In addition, AMPK activation was shown to be inversely correlated with GLI1 protein expression. Silencing AMPK expression with siRNA increased GLI1 expression, whereas activation of AMPK by metformin decreased GLI1 expression in 22Rv1 cells (Figure 3C). This indicates that metformin regulates Hh signaling, probably through AMPK signaling.

### 2.4. Combination of Metformin and GANT61 (GLI-ANTagonist 61) Synergistically Decreases PCa Cell Growth

**Figure 4:**
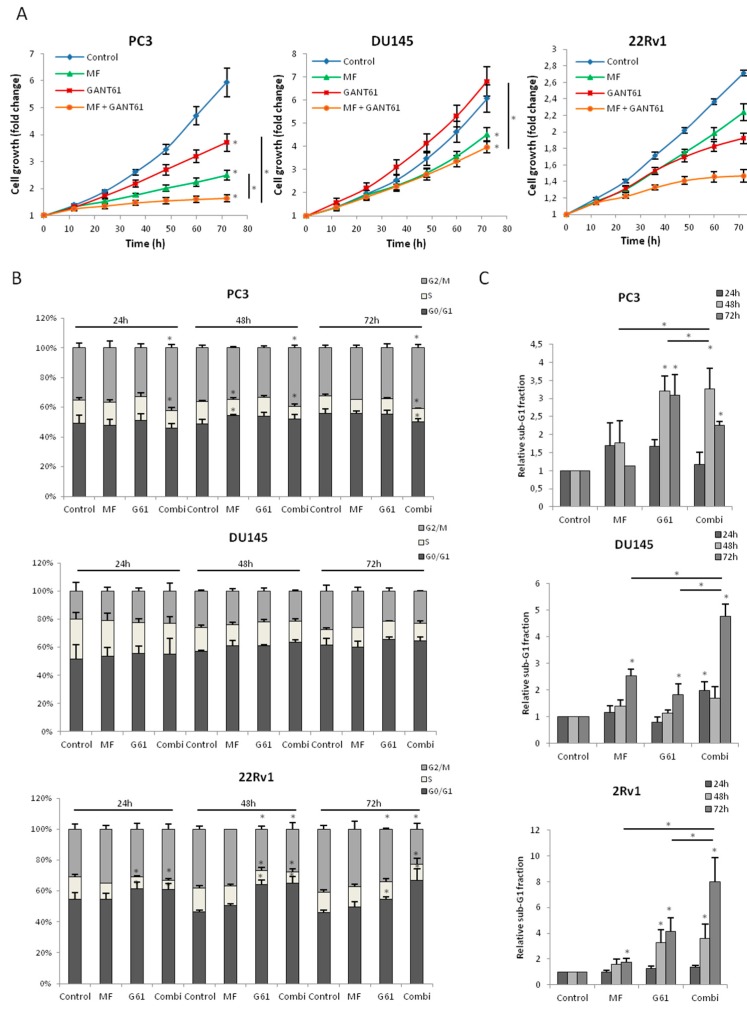
Combination of metformin and GANT61 (GLI-ANTagonist 61) in PCa cells (**A**) Cell growth; (**B**) cell cycle distribution and (**C**) sub-G1 population after treatment with metformin (5 mM), GANT61 (10 µM) or combination; (**B**,**C**) Cells were fixed at 24, 48 and 72 h of treatment. Means ± SEM of three independent experiments. * *p* < 0.05 vs. control.G61, GANT61.

The link between AMPK and GLI1 led to the question as to whether the combination of metformin with Hh inhibitors could enhance the cytotoxic effect of the individual drugs. We have previously shown that the GLI1/2 inhibitor GANT61 significantly decreased cell survival of PC3 and 22Rv1 cells [19]. Indeed, combining metformin and GANT61 significantly decreased cell growth of all PCa cell lines, resulting in an almost complete blockage of cell growth in PC3 and 22Rv1 cells (Figure 4A). Additionally, we confirmed decreased *GLI1* gene expression in all cells treated with the drug combination (Figure S2). Cell cycle analyses revealed that the drug combination in the PC3 cells led to a G2/M-arrest after only 24 h, which persisted until 72 h of treatment (Figure 4B). This corresponds to the dramatic decrease in cell growth already observed after 24 h of treatment. The drug combination also significantly increased the sub-G1 population which peaked at 48 h (Figure 4C). In the DU145 cells, no significant cell cycle effects were observed after 24–72 h of either treatment (Figure 4B), whereas the combination treatment did significantly increase apoptosis after 72 h compared to either single agent (Figure 4C). In the 22Rv1 cells, GANT61 induced a G1-arrest after only 24 h. Metformin alone did not have a significant effect on cell cycle, however, the combination of both drugs resulted in a much more pronounced G1-arrest after 72 h of treatment compared to GANT61 alone (Figure 4B). Apoptosis was also significantly induced by both individual drugs and even more by the drug combination (Figure 4C). Moreover, we observed that both drugs and their combination induced DNA damage in all cell lines after 72 h of treatment assessed by means of γH2AX staining (Figure S3).

### 2.5. Combining Metformin and GANT61 Enhances the Effect of Ionizing Radiation In Vitro

**Figure 5:**
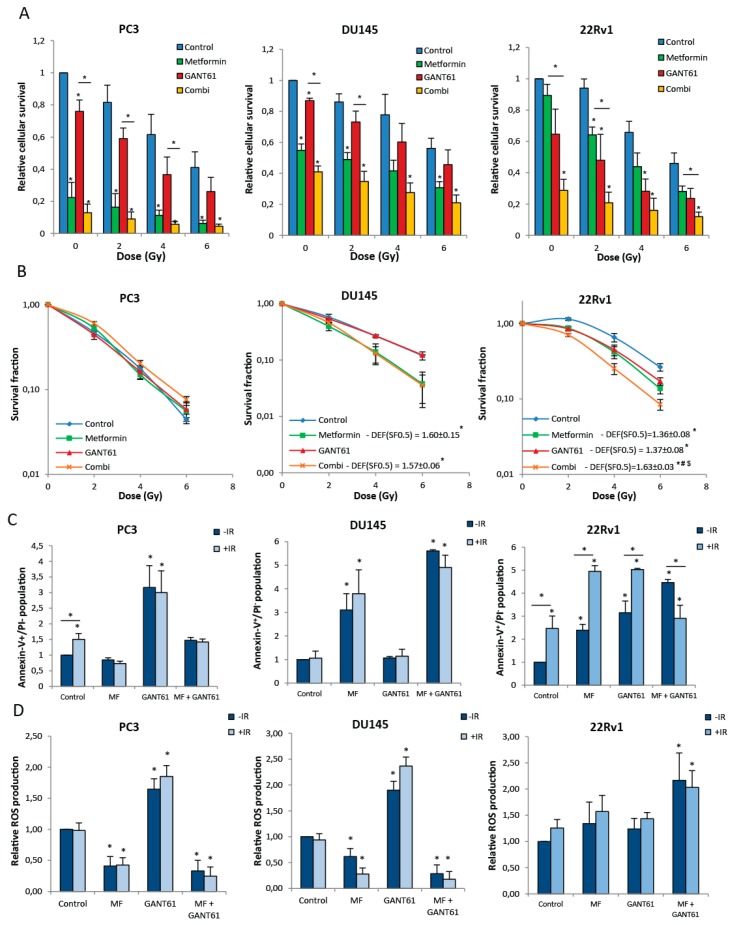
Effect of the combination of metformin and GANT61 on radiosensitivity of PCa cells. (**A**) Relative short-term cell survival 7 days after treatment with increasing doses of ionizing radiation after 72-h pretreatment with metformin, GANT61 or combination; (**B**) Clonogenic survival after 72-h treatment with metformin, GANT61 or combination prior to/during IR; (**C**) AnnexinV^+^/propidium iodide (PI)^−^ cells and (**D**) Reactive oxygen species (ROS) production at 24-h post-IR after 72-h pretreatment with metformin (5 mM), GANT61 (10 µM) or combination. Means ± SEM of three independent experiments. * *p* < 0.05 vs. control.

**Figure 6:**
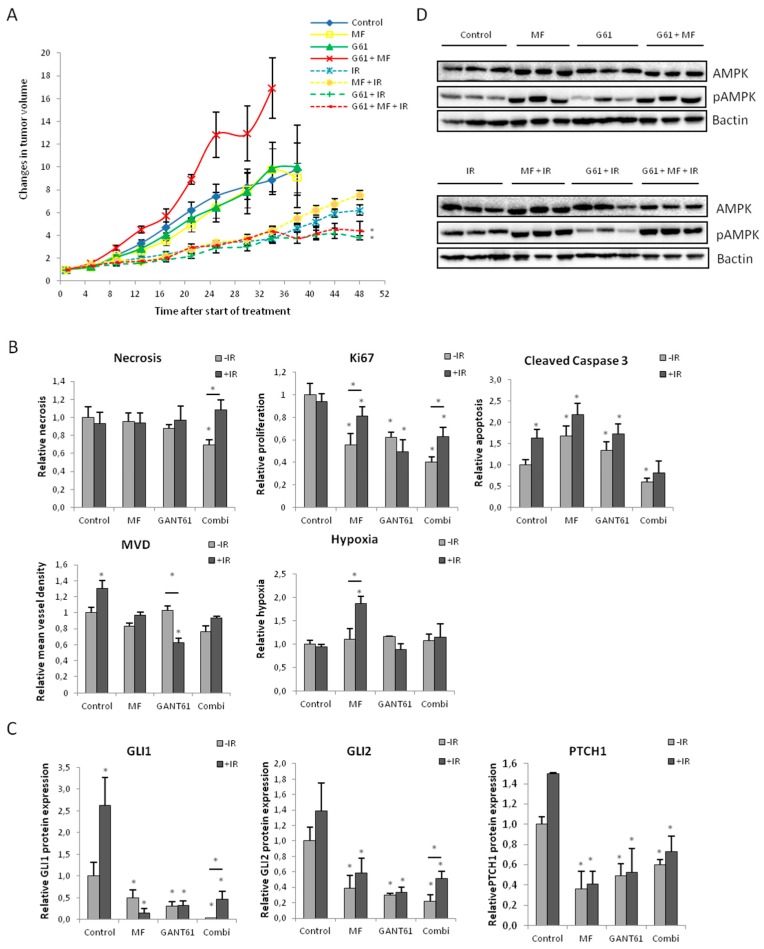
Effect of the combination of metformin and GANT61 on radiosensitivity in a 22Rv1 xenograft model. (**A**) Relative tumor growth of 22Rv1 xenograft mice treated with metformin (250 mg/kg), GANT61 (50 mg/kg) or combination. At treatment day 5, tumors were irradiated with a single dose of 6Gy (six mice/group); (**B**) Immunohistochemical analyses of necrosis, Ki67, cleaved caspase 3, mean vessel density (MVD), hypoxia and (**C**) Hedgehog (Hh) target proteins GLI1, GLI2 and PTCH1 (six mice/group, *n* ≤ 12 tumors). * *p* < 0.05 vs. control; (**D**) Protein expression of pAMPK and AMPK in 22Rv1 xenograft tumors (*n* = 3 tumors/treatment group).

Short-term survival analyses illustrated that the combination of metformin and GANT61 with ionizing radiation (IR) significantly decreased cell survival in all cell lines (Figure 5A). In the PC3 and DU145 cells, the response to IR in the combination treatment is most likely due to metformin since addition of GANT61 did not significantly decrease cell survival any further. In contrast, in the 22Rv1 cells, a significant decrease in cell survival in the combination group compared to metformin alone was observed. To assess the effect on the intrinsic radiosensitivity, clonogenic survival assays were performed. Consistent with previous results, the combination of metformin and GANT61 significantly enhanced radiosensitivity of 22Rv1 cells compared to either single agent (Figure 6B). In the PC3 cells, we did not observe an effect of the combination treatment or either single agent, whereas only metformin had a radiosensitizing effect in the DU145 cells (Figure 5B).

The combination of IR with either single agent increased apoptosis in the 22Rv1 cells. However, in the drug combination treatment, addition of IR did not further increase apoptosis; on the contrary, apoptosis was decreased in this condition indicating that other mechanisms might be important here (Figure 5C). In the PC3 and DU145 cells, apoptosis was not (further) increased upon addition of IR (Figure 5C). Furthermore, we observed that ROS were increased in the 22Rv1 cells when using the drug combination; an effect which could attribute to the increased radiosensitivity (Figure 5D).

### 2.6. Combination of Metformin and GANT61 Stimulates Tumor Growth In Vivo

Since the drug combination enhanced the radiosensitivity of 22Rv1 cells in vitro, we used a 22Rv1 xenograft model to assess the effect of the drug combination in vivo. Surprisingly, although we observed a robust decrease of cell proliferation in vitro, the combination of GANT61 and metformin appeared to be pro-proliferative in vivo*.* Metformin and GANT61 alone did not have any effect on tumor growth (Figure 6A). To investigate why the combination of both drugs resulted in a paradoxical effect, we looked deeper into the molecular changes that occurred in these tumors. Tumors treated with the drug combination were less necrotic and had fewer apoptotic cells compared to control tumors or those treated with either single agent (Figure 6B). In addition, these tumors contained fewer blood vessels compared to controls or the GANT61-treated tumors. No changes in hypoxia were observed. Although the drug combination increased tumor growth, this was not reflected by Ki67 expression, which was significantly lower in these tumors compared to controls and either single agent (Figure 6B). This implies that the growth stimulatory effect of the drug combination is more likely due to pro-survival effects rather than effects on proliferation.

As for the combination with IR, metformin did not increase radiosensitivity of 22Rv1 tumors, while GANT61 did result in enhanced radiosensitivity. The combination of metformin and GANT61 with IR also significantly decreased tumor growth, however this was most likely attributed to the effect of GANT61 (Figure 6A). As previously shown [19], the GANT61-induced radiosensitization is attributed to decreased proliferation and increased apoptosis. In the tumors treated with the drug combination, the radiosensitizing effect can also, at least partially, be ascribed to effects on proliferation. In contrast, the amount of apoptosis is lower in these tumors compared to controls or tumors treated with a single agent, but we observed more necrosis in the tumors treated with the combined modality, which could also attribute to the decreased tumor growth (Figure 6B).

Finally, we verified whether both drugs reached their target in the tumors to verify their specificity in vivo. Immunohistochemical analyses of GLI1, GLI2 and PTCH1 demonstrated that GANT61 was able to specifically inhibit Hh signaling in the tumor (Figure 6C). We also provide evidence that metformin targets AMPK signaling as phospho-AMPK (pAMPK) was significantly increased in all tumor of metformin-treated mice (Figure 6D). Additionally, we showed that the link between metformin and Hh signaling also exists in vivo. Both metformin also significantly decreased protein expression of GLI1, GLI2 and PTCH1 (Figure 6C).

## 3. Discussion

Contradicting findings have been reported in PCa patients regarding an association between metformin use in diabetic PCa patients and biochemical recurrence (BCR) and metastasis. Several studies have shown that metformin had no effect on BCR-free survival of PCa patients after radical prostatectomy [20,21,22,23]. In contrast, a retrospective study by He et al. [24] did find a beneficial effect of metformin on overall survival (OS) of PCa patients treated with radical prostatectomy [24]. In a study by Spratt et al. [25] the authors specifically looked at PCa patients treated with external-beam radiotherapy (EBRT) and found a significant positive correlation between metformin use and BCR-free survival and OS [25]. These studies demonstrate that metformin might be an important treatment option for PCa patients, especially in the combination setting with EBRT.

In this study, we first evaluated the effect of metformin on cell growth and radiosensitivity of PCa cells. In line with a previous study by Sahra et al. [4], we found that metformin (5 mM) decreased cell growth of all cell lines, which coincided with reduced protein levels of cyclin D1. The fact that we could only observe a significant inhibition of cell growth at high doses of metformin is in accordance with multiple studies using different tumor cell types [4,8,26,27,28]. Several preclinical studies have already investigated the effect of metformin on the radiosensitivity of different tumor types and multiple mechanisms have been associated with the enhanced radiation sensitivity after metformin treatment [8,9,10,11,12]. Here, a radiosensitizing effect of metformin was observed in the DU145 and 22Rv1 cells, but not in the PC3 cells. This lack of radiosensitization in the PC3 cells could be a result of the higher sensitivity of PC3 cells to metformin leading to the absence of any additional effect upon IR. Data also suggest that metformin may enhance radiation response specifically in certain genetic backgrounds (p53, liver kinase B1 (LKB1)), but more research is needed to gain better insight in the specific interactions between metformin and important oncogenic and tumor suppressor genes [28].

We show that metformin also interacts with the Hh signaling pathway, specifically by decreasing gene and protein expression of the Hh target genes *GLI1* and *PTCH1*. Similar interactions were described recently in breast [13] and pancreatic [14] cancer cells. Our data demonstrate that this interaction is mediated by the activation of AMPK signaling, which is in line with the data of Nakamura et al. [14]. Accordingly, two recent studies have shown the AMPK phosphorylates GLI1, which results in the proteasomal degradation of GLI1 [29,30]. On top of that, we demonstrate that metformin also decreased Hh signaling in vivo. Interestingly, we observed a differential inhibition of Hh signaling in the three PCa cell lines at the gene level. In the PC3 and 22Rv1 cells, metformin significantly inhibited *GLI1* and *PTCH1* gene expression, whereas in the DU145 cells, metformin strongly inhibited *GLI2* gene expression in addition to inhibiting *GLI1* and *PTCH1*. Nevertheless, we did not observe a different phenotype in these cell lines in response to metformin treatment.

In a previous study by our group, we demonstrated that Hh inhibition increased the intrinsic radiosensitivity of 22Rv1 cells and not of PC3 and DU145 cells, which appeared to be dependent on functional p53 signaling [19]. Furthermore, it seems unlikely that the effect of metformin on radiosensitivity is through its effects on Hh signaling, as we have shown that metformin regulates Hh signaling in an AMPK-dependent manner, whereas it increases radiosensitivity of PCa cells independent of AMPK activation. Therefore, Hh inhibition and metformin both increase radiosensitivity of PCa cells but in a different manner.

This interesting interaction between metformin and Hh signaling suggests that the combination of metformin with Hh inhibition has the potential to further enhance the cytotoxic and/or radiosensitizing effect of either single agent. To our best knowledge, this combination has never been tested before. Our results show that this combination treatment is highly effective against PCa proliferation in vitro. This was associated with an increase of apoptosis and the induction of DNA damage in all cell lines. Additionally, we observed G2/M-arrest after just 24 h in the PC3 cells, these being the most sensitive to this drug combination. In the 22Rv1 cells, the cells treated with the drug combination experienced G1-arrest, which was at least partially ascribed to GANT61. In contrast with the high cytotoxicity of the drug combination observed in vitro, combining metformin and GANT61 appeared to be pro-proliferative in a 22Rv1 xenograft mouse model. Strangely, the amount of proliferation in these tumors was significantly decreased compared to control or either single agent, suggesting that the increased tumor growth was more likely attributed to decreased apoptosis and necrosis.

Our data also suggest that the combination of metformin and GANT61 only increases radiosensitivity of cells that are sensitized by both individual drugs, as was the case with the 22Rv1 cells. The PC3 cells were not radiosensitized by either drug or the combination, whereas the DU145 cells were only sensitized by metformin and not by GANT61 due to its p53 mutation [19], resulting in no extra benefit upon drug combination. In the 22Rv1 cells we observed a radiosensitizing effect of both metformin and GANT61 which was further enhanced by the drug combination. This was not the case in the in vivo situation, where we only observed a radiosensitizing effect of GANT61 and no additive effect on radiosensitivity by metformin. In line with our previously published data, the GANT61-induced radiosensitization was associated with decreased proliferation and increased cell death [19]. In tumors treated with the drug combination and IR, the amount of necrosis was significantly increased. One potential reason for the lack of radiosensitization of metformin in vivo might be the dosage of metformin used. In vitro, high-doses of metformin (5 mM) are needed to induce cytotoxic and radiosensitizing effects. These high levels of metformin are typically not achieved in an in vivo situation. We used 250 mg/kg intraperitoneally, which is also considered quite high compared to the typical metformin dosing in diabetic patients which lies around 30 mg/kg orally. However, considering the interspecies differences between mice and humans, the Food and Drug Administration (FDA) applies a standard scaling factor of 12.3 [31] which makes our in vivo dosage used definitely acceptable.

Several Smoothened (SMO) inhibitors are currently in the early stages of clinical investigation in PCa patients. Despite the high efficacy of SMO inhibition in patients with basal cell carcinoma and medulloblastoma, which are characterized by the presence of Hh mutations, the efficacy of these inhibitors in ligand-dependent Hh-activated cancer types such as PCa has not yet been established. Early phase clinical trials demonstrated little or no responsiveness to Hh inhibition in these tumor types so far. Multiple clinical trials are currently ongoing to investigate the potential of Hh inhibition as monotherapy or in combination with hormonal therapy in PCa [32]. The use of Hh inhibitors more downstream the signaling cascade has not yet been investigated in patients. Based on our results, the combination of GANT61 with radiotherapy might also be a promising strategy to test in the clinic [19].

Although the combination of metformin and GANT61 therapy appeared very effective in an in vitro situation, the interaction of metformin and GANT61 in the in vivo setting resulted in a paradoxical (pro-survival) effect. This suggests that the tumor microenvironment could play an important role in the anti-tumor activity of drug combinations in vivo. We have previously shown that Hh inhibition indeed also targets the stromal compartment of the tumor and that this results in an increased efficacy of radiotherapy and Hh inhibition in two PCa xenograft models [19]. This was in line with other reports indicating that the tumor-associated stroma is also influenced by Hh inhibition and this might have an impact on the tumor cells [33,34]. Additionally, metformin has also been shown to influence the tumor microenvironment. A study by Martin et al. [35] has found that metformin induced vascular endothelial growth factor A (VEGF-A) expression in a BRAF-driven melanoma tumor model. This resulted in increased angiogenesis and accelerated tumor growth [35]. Although we also observed an enhanced tumor growth in the combination group, we did not observe any changes in microvessel density in our study.

This observation highlights the importance of testing drug interactions in an in vivo setting. The effect of drug interactions is often overlooked in preclinical studies, resulting in the failure of many novel medications in clinical trials. Therefore, more research into the interaction between Hh inhibition and metformin should be performed in multiple tumor models to guarantee the safety of Hh inhibition in diabetic patients using metformin.

## 4. Materials and Methods

### 4.1. Cell Culture and Drug Exposure

The androgen-unresponsive PCa cell lines PC3 and DU145 were obtained from the American Type Culture Collection (ATCC; Manassas, VA, USA). The PC3 cells were grown in minimal essential medium (MEM, Life Technologies, Carlsbad, CA, USA) supplemented with 10% fetal bovine serum (FBS; Life Technologies). The DU145 cells were cultured in MEM (Life Technologies) supplemented with 10% FBS, 1% sodium pyruvate (Life Technologies) and 1% non-essential amino acids (Life Technologies). The androgen-responsive 22Rv1 cells (European Collection of Cell Cultures, ECACC, Salisbury, UK) were cultured in RPMI 1640 medium without phenol red (Sigma-Aldrich, St. Louis, MA, USA), supplemented with 10% FBS, 1% l-glutamine and 1% 4-(2-hydroxyethyl)-1-piperazineethanesulfonic acid (HEPES) buffer (Life Technologies). All cells were maintained at 37 °C in a humidified incubator with 5% CO_2_/95% O_2_ atmosphere.

Stock solutions of metformin (Sigma-Aldrich) were prepared in sterile water or saline for in vitro and in vivo experiments, respectively. For in vitro experiments, stock solutions of GANT61 were prepared in dimethyl sulfoxide (DMSO; Adipogen, San Diego, CA, USA). For the in vivo experiment, GANT61 (Tocris, Bristol, UK) was dissolved in 100% EtOH and was further dissolved in saline (9:1 saline:EtOH). Control conditions were treated with the corresponding drug solvent.

### 4.2. Cell Growth and Survival

Cells were seeded in a 96-well plate at a density of 2.5–45 × 10^4^ cells per well and treated for 72 h with different concentrations of the inhibitors. Cell growth was assessed using the Incucyte Zoom system (Essen BioScience, Ann Arbor, MI, USA). Short-term survival assays were performed by pretreating the cells with GANT61 (10 µM) and metformin (5 mM) for 72 h followed by IR (2, 4, or 6 Gy). After 24 h, fresh medium was added and cell survival was assessed 7 days thereafter by means of sulforhodamine B (SRB) assay [36].

### 4.3. Quantitative Real-Time Polymerase Chain Reaction (qPCR)

RNA isolation and quantitative Polymerase Chain Reaction (qPCR) reactions were performed as previously described [19]. Primer sequences for glyceraldehyde 3-phosphate dehydrogenase (GAPDH), PTCH1, GLI1 and GLI2 are enlisted in Supplemental Table S1. Gene expression was calculated as expression per 100,000 copies of the household gene *GAPDH*.

### 4.4. Immunoblot Analysis

Immunoblotting was performed as done before [19]. Primary antibodies against ACC (#3662, 1:1000), phospho-acetyl-CoA carboxylase (pACC) (#3661, 1:500), AMPK (#2532, 1:1000), pAMPK (#2535, 1:500), GLI1 (#2534, 1:500) from Cell Signaling Technologies (Beverly, MA, USA), cyclin D1 (CCND1) (sc-8396, 1:200) and PTCH1 (sc-6149, 1:200) from Santa Cruz (Dallas, TX, USA) and GLI2 (600-401-845, 1:1000) from Rockland Immunochemicals (Limerick, PA, USA) were used. Β-actin (Cell Signaling Technologies, #4967, 1:1000) was used as loading control. An enhanced chemiluminescence detection system (Perkin Elmer, Waltham, MA, USA) was used to visualize immune-reactive proteins using Fujifilm LAS-3000 mini camera (Fujifilm, Germany). Protein expression was quantified using ImageJ 1.50.

### 4.5. Flow Cytometry

Apoptotic cell populations (AnnexinV^+^/PI^−^),DNA damage and cell cycle distribution were measured as previously described [19]. Reactive oxygen species (ROS) were detected using 2′,7′-dichlorodihydrofluorescein diacetate [37]. FACSVerse Flow cytometer (BD Biosciences, Franklin Lakes, NJ, USA) was used for flow cytometric analysis.

### 4.6. Colony Formation

Clonogenic formation assays were performed as previously described [19]. After 72-h drug treatment, cells were seeded at low density and irradiated with 2, 4 and 6 Gy using a Baltograph (Balteau NDT, Hermalle-sous-argenteau, Belgium) or mock irradiated. Fresh medium was added 16 h post-irradiation. After 11–21 days post-irradiation, cells were fixed with 2.5% glutaraldehyde in phosphate buffered saline (PBS) and stained with 0.4% crystal violet. The colonies containing ≥50 cells were counted with ColCount (Oxford Optronix, Oxford, UK). Survival fractions normalized for drug-induced toxicity. Dose-enhancement factor (DEF) was calculated as the ratio of the dose needed for the control cells to the dose needed for the treated cells to reach a survival fraction of 0.5 (DEF0.5).

### 4.7. Animal Experiments

Animal experiments were approved by the ethics committee of KU Leuven (P131/2014). Male NMRI Nu/Nu mice (Janvier, France) were inoculated in both flanks with 2 × 10^6^ 22Rv1 cells in 100:100 µL medium/Matrigel (VWR, Radnor, PA, USA). Once tumors reached a volume of 150 mm^3^, mice were treated by intraperitoneal injection with solvent (9:1 saline/EtOH), metformin (250 mg/kg, every day), GANT61 (50 mg/kg every other day) or the combination of both drugs for 7 weeks. At day 5 of drug treatment, tumors were irradiated with a single dose of 6 Gy. During the entire treatment period, tumor growth was followed by 2-weekly caliper measurements and tumor volumes were calculated (V = (length × width × height) × π/6). In addition, the body weight of the mice was monitored to assess potential treatment toxicity. Mice were euthanized at the end of drug treatment or when tumors reached the maximum ethically permitted volume of 2 × 10^3^ mm^3^. Thirty min before euthanasia, pimonidazole was intraperitoneally injected. Afterwards, tumors were excised and half of the tumor was fixed in formalin and embedded in paraffin for immunohistochemical analysis and the other half was snap-frozen for protein analysis.

### 4.8. Immunohistochemistry

Immunohistochemistry for Ki67, cleaved caspase3, pimonidazole, cluster of differentiation 31 (CD31), GLI1, GLI2 and PTCH1 was performed as previously described [19]. Protein expression was quantified using ImageJ.

### 4.9. Statistical Analysis

One-way ANOVA with Tukey’s multiple comparison test or a two-tailed student’s *t*-test were used for the in vitro experiments. For the in vivo experiment, a Kolmogorov–Smirnov method was used to test for normality. Thereafter, either a two-tailed student’s *t*-test was used when the data were normally distributed with equal variance or nonparametric analysis using the Mann–Whitney rank-sum test in other conditions. All statistical tests were performed using the software package Statistica 12 (StatSoft Inc., Tulsa, OK, USA). A *p*-value of <0.05 was considered statistically significant.

## Supplementary Materials

Supplementary materials can be found at www.mdpi.com/1422-0067/18/2/399/s1.

## Abbreviations

ACC	Acetyl-CoA carboxylase
AMP	Adenosine monophosphate
AMPK	AMP-activated protein kinase
CCND1	Cyclin D1
CD31	Cluster of differentiation 31
qPCR	Quantitative Polymerase Chain Reaction
DEF	Dose-enhancement factor
DMSO	Dimethyl sulfoxide
EBRT	External-beam radiotherapy
ECACC	European Collection of Cell Cultures
FBS	Fetal bovine serum
GANT61	GLI-ANTagonist 61
GAPDH	Glyceraldehyde 3-phosphate dehydrogenase
GLI	Glioma-associated oncogene homolog
HEPES	4-(2-hydroxyethyl)-1-piperazineethanesulfonic acid
Hh	Hedgehog
IC_50_	Half maximal inhibitory concentration
IR	Ionizing radiation
LKB1	Liver kinase B1
MEM	Minimal essential medium
MF	Metformin
mTOR	Mammalian target of rapamycin
MVD	Microvessel density
OS	Overall survival
pACC	Phospho Acetyl-CoA carboxylase
pAMPK	Phospho AMP-activated protein kinase
PBS	Phosphate buffered saline
PI	Propidium iodide
PTCH1	Patched 1
PCa	Prostate cancer
ROS	Reactive oxygen species
SEM	Standard error of means
siRNA	Silencing RNA
SMO	Smoothened
VEGF-A	Vascular endothelial growth factor A
