# Supplementary material for: The Effect of Metformin and GANT61 Combinations on the Radiosensitivity of Prostate Cancer Cells
Source: Int J Mol Sci. 2017 Feb 13;18(2):399. doi: 10.3390/ijms18020399 (PMC5343511; doi:10.3390/ijms18020399)
Supplement: Supplementary file 1 [file ijms-18-00399-s001.pdf]

Supplementary Materials: The Effect of Metformin and GANT61 Combinations on the Radiosensitivity of Prostate Cancer Cells

Annelies Gonnissen, Sofie Isebaert, Chad M. McKee, Ruth J. Muschel and Karin Haustermans

Table S1. Forward and reverse primer sequences used for quantitative polymerase chain reaction (qPCR).

Gene	Forward 5′–3′	Reverse 5′–3′
GAPDH	CCATCTTCCAGGAGCGAG	TGAAGACGCCAGTGGAC
PTCH	AAACAGGTTACATGGATCAGATAATAG	CCCTTCCCAGAAGCAGT
GLI1	AATGCTGCCATGGATGCTAGA	GAGTATCAGTAGGTGGGAAGTCCATAT
GLI2	GCCCTCACCTCCATCAAT	TGTTCTGGTTGGTGTCACT

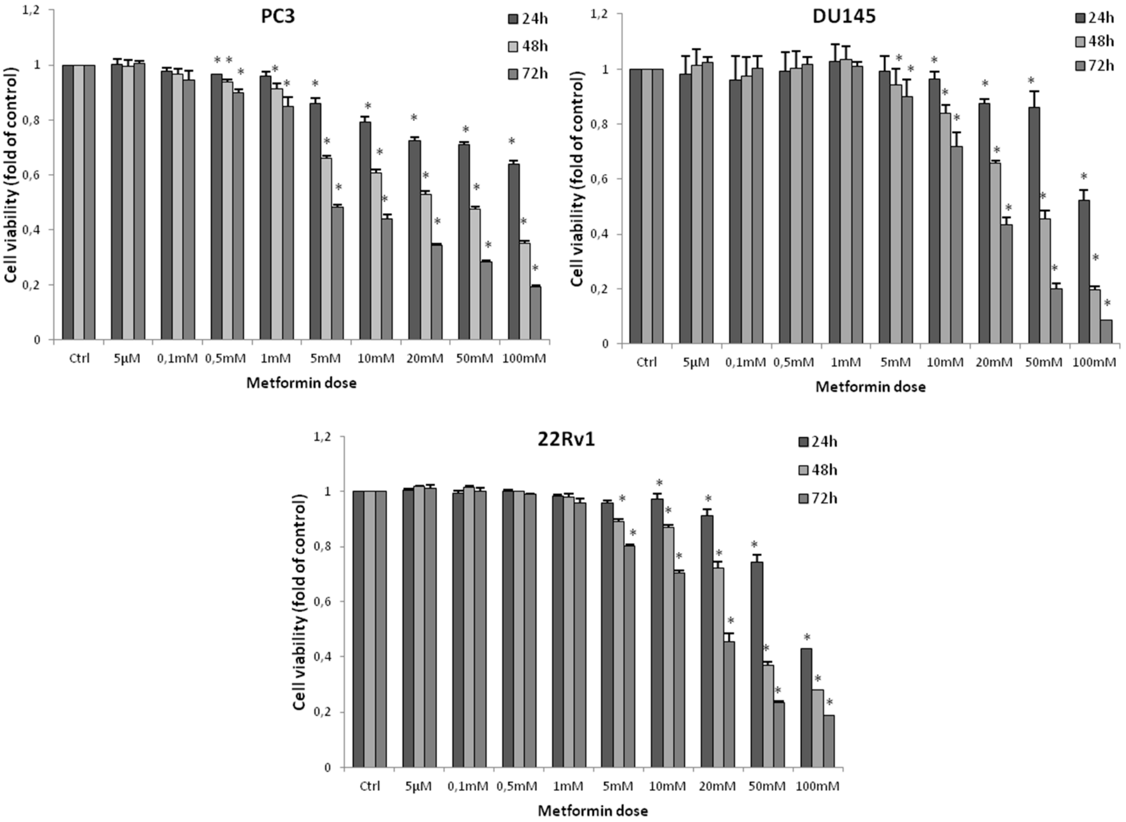

Figure S1. Cell viability (fold of control) after metformin treatment. Means ± standard error of the means (SEM) of two independent experiments. * $p < 0.05$ vs. control.

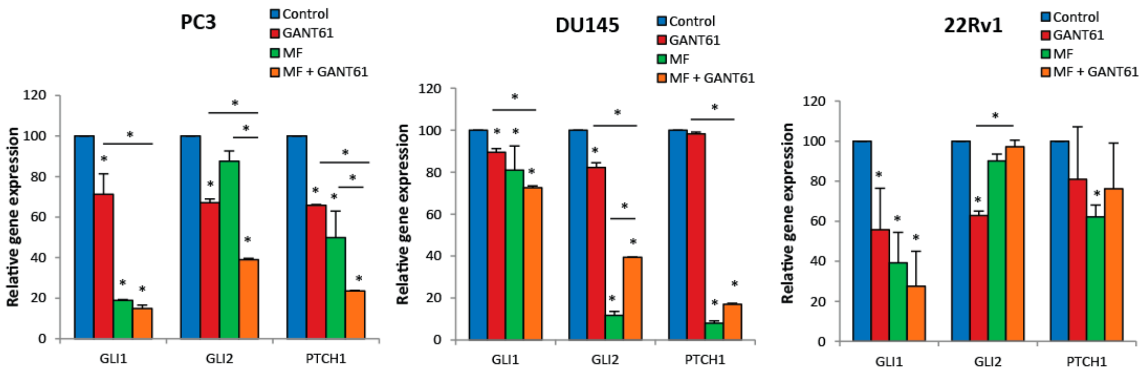

Figure S2. Hedgehog (Hh) gene expression after metformin (MF), GANT61(GLI-ANTagonist 61) or combined treatment. *GLI1*, *GLI2* and *PTCH1* gene expression in PC3, DU145 and 22Rv1 cells after 72-h metformin (5 mM), GANT61 (10 µM) or combination treatment. Means ± SEM of two independent experiments performed in triplicate. * $p < 0.05$ vs. control. GLI1, glioma-associated oncogene homolog 1; GLI2, glioma-associated oncogene homolog 2; PTCH1, patched 1.

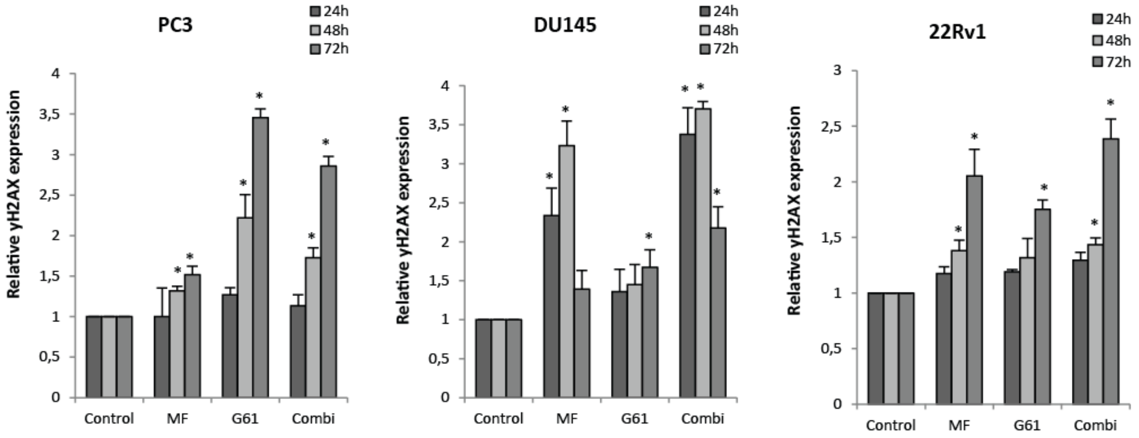

Figure S3. Effect of metformin and GANT61 (G61) on induction of DNA damage and phosphorylation of H2AX after 24-, 48- and 72-h drug treatment in PC3, DU145 and 22Rv1 cells. Cells were treated for 72 h with metformin (5 mM), GANT61 (10 µM) or combination and cells were fixed at 24, 48 and 72 h of treatment. Means ± SEM of three independent experiments. * $p < 0.05$ vs. control.
